# Anti‐TNF treatment negatively regulates human CD4^+^ T‐cell activation and maturation in vitro, but does not confer an anergic or suppressive phenotype

**DOI:** 10.1002/eji.201948190

**Published:** 2019-12-03

**Authors:** Giovanni A. M. Povoleri, Sylvine Lalnunhlimi, Kathryn J. A. Steel, Shweta Agrawal, Aoife M. O'Byrne, Michael Ridley, Shahram Kordasti, Klaus S. Frederiksen, Ceri A. Roberts, Leonie S. Taams

**Affiliations:** ^1^ Centre for Inflammation Biology and Cancer Immunology (CIBCI), Dept of Inflammation Biology, School of Immunology & Microbial Sciences King's College London London UK; ^2^ Comprehensive Cancer Centre, School of Cancer and Pharmaceutical Sciences King's College London London UK; ^3^ Global Drug Discovery Diabetes Research Novo Nordisk A/S Måløv Denmark

**Keywords:** adalimumab, CD4^+^ T cells, CyTOF, interleukin‐10, TNF inhibitor

## Abstract

TNF‐blockade has shown clear therapeutic value in rheumatoid arthritis and other immune‐mediated inflammatory diseases, however its mechanism of action is not fully elucidated. We investigated the effects of TNF‐blockade on CD4^+^ T cell activation, maturation, and proliferation, and assessed whether TNF‐inhibitors confer regulatory potential to CD4^+^ T cells. CyTOF and flow cytometry analysis revealed that in vitro treatment of human CD4^+^ T cells with the anti‐TNF monoclonal antibody adalimumab promoted IL‐10 expression in CD4^+^ T cells, whilst decreasing cellular activation. In line with this, analysis of gene expression profiling datasets of anti‐TNF‐treated IL‐17 or IFN‐γ‐producing CD4^+^ T cells revealed changes in multiple pathways associated with cell cycle and proliferation. Kinetics experiments showed that anti‐TNF treatment led to delayed, rather than impaired T‐cell activation and maturation. Whilst anti‐TNF‐treated CD4^+^ T cells displayed some hyporesponsiveness upon restimulation, they did not acquire enhanced capacity to suppress T‐cell responses or modulate monocyte phenotype. These cells however displayed a reduced ability to induce IL‐6 and IL‐8 production by synovial fibroblasts. Together, these data indicate that anti‐TNF treatment delays human CD4^+^ T‐cell activation, maturation, and proliferation, and this reduced activation state may impair their ability to activate stromal cells.

## Graphical abstract

Through deep phenotyping using CyTOF and functional assays we have shown that TNF inhibition using adalimumab altered the phenotype and function of multiple populations of human CD4+ T‐cells, leading to an overall decreased pro‐inflammatory potential. These results provide insight into the anti‐TNF mechanism of action in human CD4+ T‐cells.

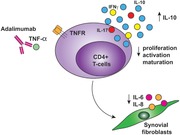

## Introduction

Rheumatoid arthritis (RA) is a chronic inflammatory condition, characterised by synovial inflammation and hyperplasia, as well as autoantibody production in the majority of cases. The disease can lead to debilitating bone and cartilage destruction, which can result in severe disability and is associated with premature mortality 1.

The pathophysiology of the disease is still not fully understood. It is believed that CD4^+^ T cells play a key role in RA pathogenesis; however, therapies specifically targeting CD4^+^ T cells have not entered clinical use due to limited efficacy 2. Nonetheless, immunological cross‐talk between CD4^+^ T cells and other immune or stromal cells is an important factor for established RA disease 3: CD4^+^ T cells can stimulate monocytes, macrophages, and fibrobrast‐like synoviocytes to secrete various pro‐inflammatory cytokines 4. In turn, upon encounter with synovial monocytes, macrophages, or fibroblasts, CD4^+^ T cells can secrete multiple cytokines including IFN‐γ, IL‐17, and TNF 5, 6, 7, 8, which can overcome negative regulators of inflammation such as IL‐10, thus creating a predominant pro‐inflammatory environment 9.

Multiple pro‐inflammatory cytokines detected in RA synovium have been identified as therapeutic targets including IL‐1, IL‐6, and TNF 10. Several TNF‐inhibitors have been engineered for the treatment of RA, amongst them is adalimumab (ADA), a fully human anti‐TNF monoclonal antibody 11. TNF‐inhibitors have shown demonstrable clinical success, however, loss of efficacy over time is observed and approximately one‐third of patients are or become unresponsive to treatment. The reasons for this remain unclear 12 and it is currently not possible to reliably predict which patients will not respond 13, 14. A better understanding of the mechanisms of action of TNF inhibition may help shed light on these issues.

Our lab recently demonstrated that adalimumab treatment led to an increase in the proportion of CD4^+^ T cells that express the anti‐inflammatory cytokine IL‐10, both ex vivo and in vitro 15, 16. This increase occurred in T cell populations that co‐expressed pro‐inflammatory cytokines such as IFN‐γ, IL‐17, TNF‐α, or GM‐CSF, and was independent of Tregs. In this study, we further investigated the effects of TNF‐blockade on CD4^+^ T‐cell activation, maturation, proliferation and assessed whether TNF‐inhibitors may confer regulatory potential to CD4^+^ T cells.

## Results

### ADA treatment leads to IL‐10 induction in multiple CD4^+^ T cell subsets

**Figure 1:**
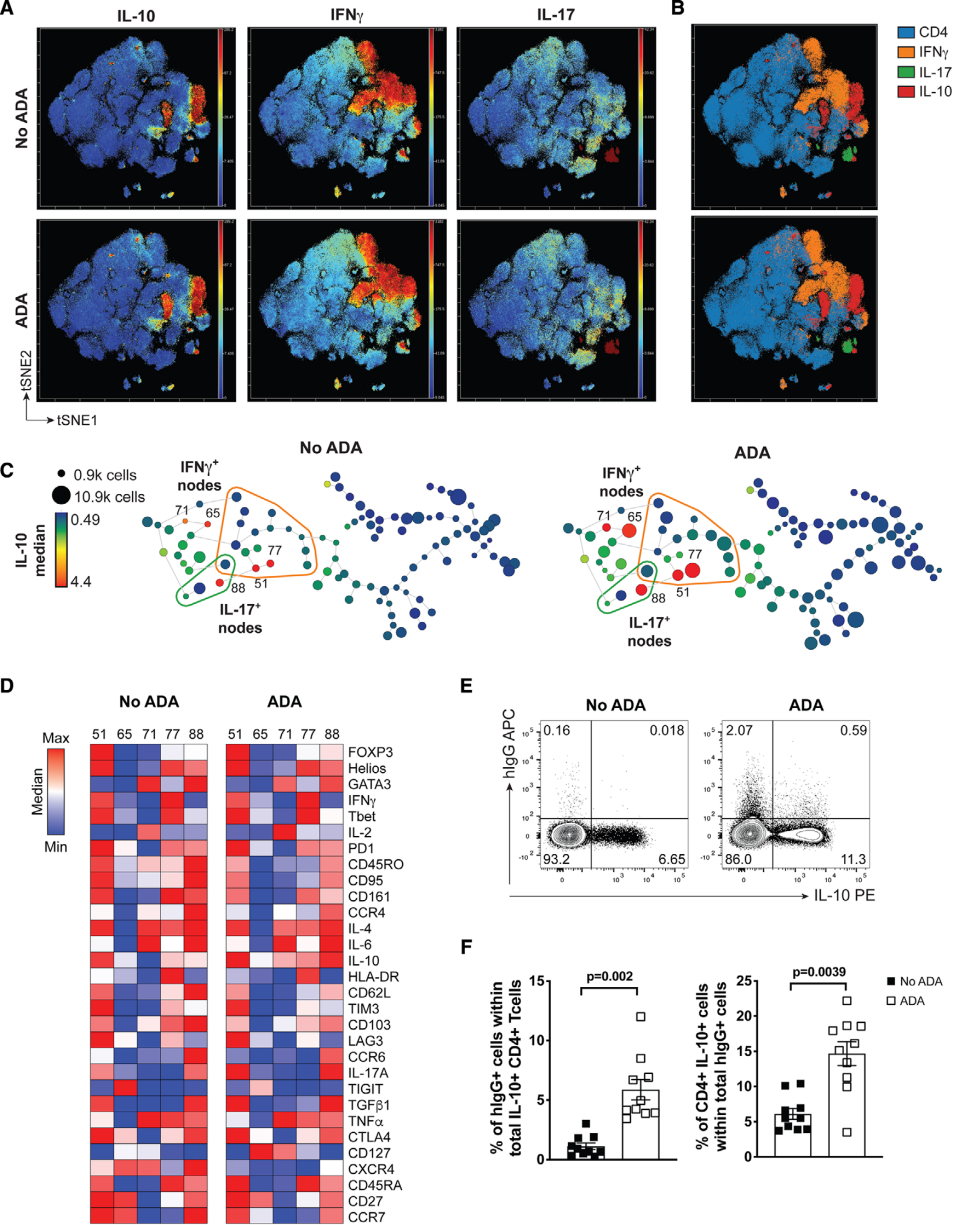
Adalimumab (ADA) treatment enhances IL‐10 production in multiple subsets of cytokine producing CD4^+^ T cells. (A) viSNE plots of CyTOF data from CD4^+^ T cells stimulated for 3 days with aCD3/CD28 mAb, and 3 h of PMA/Ionomycin, in the absence (top row) or presence (bottom row) of ADA; cells were clustered using surface and intracellular markers. Shown are heatmaps for expression of indicated markers. (B) Overlay of IL‐10^+^ (red), IFN‐γ^+^ (orange), and IL‐17^+^ (green) T cells on viSNE map of CD4^+^ T cells (blue) showing the overlap of cells either expressing IL‐10 alone or in combination with other cytokines in the absence or presence of ADA; (C) 2D minimum spanning tree (SPADE) derived from viSNE analysis showing population nodes of CD4^+^ T cells either cultured in the absence (left panel) or presence (right paneI) of ADA. Node size represents cell number, and colour IL‐10 median intensity. Grouped together are IL‐17^+^ (circled in green) and IFN‐γ^+^ (circled in orange) nodes. Numbered nodes in C represent IL‐10^+^ nodes identified from Supporting Information Fig. 1A. (D) Correlation matrix showing median expression of markers from IL‐10^+^ nodes from 1C and Supporting Information Fig. 1A. (E) Representative flow cytometry plot showing frequencies of CD4^+^ T cells stained for IL‐10 and hIgG at day 3 of stimulation with aCD3/CD28 mAb, in the absence or presence of ADA, and (F) cumulative data showing the frequency of hIgG^+^ cells within total IL‐10^+^ CD4^+^ T cells (left) and IL‐10^+^ CD4^+^ T cells within total hIgG^+^ cells (right). Bars show mean ± SEM of results from six independent experiments using *n* = 10 distinct donors. Data analysed by two‐tailed paired Wilcoxon test.

To evaluate the effect of ADA treatment on IL‐10 induction in pro‐inflammatory populations of CD4^+^ T cells, we performed mass cytometry (CyTOF) to obtain an unbiased, single‐cell, multi‐dimensional analysis of IL‐10 expression among different populations of cytokine producing cells. CD4^+^ T cells were stimulated with anti‐CD3 and anti‐CD28 (aCD3/CD28) mAb for 3 days in the absence or presence of 1 µg/mL of ADA followed by 3 h of PMA/ionomycin stimulation in the presence of GolgiStop. Visual stochastic neighbour embedding (viSNE) was used to create a map of CD4^+^ T cells; cells were automatically arranged along t‐distributed stochastic neighbour embedding (t‐SNE) axes based on phenotypic similarity of each cell 17. This unsupervised viSNE analysis visualised multiple clusters of IL‐10, IFN‐γ, and IL‐17 producing CD4^+^ T cells (Fig. 1A). While ADA treatment did not affect the expression of IFN‐γ and IL‐17, an increase in intensity and density of IL‐10^+^ clusters was observed (Fig. 1A). Furthermore, our analysis confirmed our previous data 16 that ADA affected IL‐10 expression in multiple clusters of CD4^+^ T cells, including in clusters of IFN‐γ and IL‐17 expressing cells (Fig. 1B). To identify these distinct populations (or nodes) of cytokine producing T cells, based on the differential expression of the markers used for the tSNE analysis, we performed a spanning‐tree progression analysis of density‐normalised events (SPADE) 18. SPADE analysis resolved CD4^+^ T cells into 100 sub‐populations, each characterized by a different expression profile (Fig. 1C).

Marker enrichment modelling (MEM) 19 was performed to distinguish the IL‐10 producing populations by quantifying protein enrichment values within the SPADE nodes (Supporting Information Fig. 1A). MEM analysis allowed the identification of five IL‐10 producing populations with an IL‐10 MEM enrichment score ≥ +2.5 in the ADA condition (Supporting Information Fig. 1A, indicated by node numbers in bold). To increase resolution and better discriminate the different IL‐10 producing sub‐populations, viSNE and SPADE analysis were also run on gated CD4^+^CD45RO^+^ cells (Supporting Information Fig. 1B–E), revealing 50 sub‐populations (Supporting Information Fig. 1E). MEM analysis of memory CD4^+^ T cells identified 12 IL‐10 producing populations (Supporting Information Fig. 1F). These analyses confirmed that culturing CD4^+^ T cells with ADA increased the expression of IL‐10 in multiple distinct CD4^+^ T cell subsets, including those expressing IL‐17 and IFN‐γ. Furthermore, ADA treatment led to expansion of IL‐10 producing populations (e.g. CD4^+^ T cell nodes 65 and 77, Fig. 1C), while induction of IL‐10 in some cells was revealed by focusing the clustering analysis on the “enriched” CD4^+^CD45RO^+^ cells (e.g. nodes 16 and 38, Supporting Information Fig. 1E). Finally, MEM analysis revealed that ADA treatment did not result in a dramatic change in the broad phenotype of IL‐10^+^ populations, as compared to IL‐10^+^ cells in cultures where no ADA was present (Fig. 1D and Supporting Information Fig. 1G).

Since adalimumab is known to bind to the transmembrane form of TNF (mTNF) 20, we sought to determine whether there was an association between IL‐10 producing cells and ADA‐bound cells. We assessed the binding of adalimumab (an IgG1 human antibody) on CD4^+^ T cells after 3 days of culture, using a fluorochrome‐conjugated mouse anti‐human IgG Fc antibody. We found little expression of mTNF on CD4^+^ T cells (Supporting Information Fig. 1H), which was reflected by only a small proportion of CD4^+^ T cells positive for surface bound human IgG in cultured samples treated with ADA for up to 3 days (Fig. 1E and Supporting Information Fig. 1I). Furthermore, when we specifically analysed the IL‐10^+^ CD4^+^ T cells, a minority (on average 5.9%) of IL‐10^+^ CD4^+^ T cells stained positive for human IgG; vice versa, on average only 14.7% of hIgG^+^ cells expressed IL‐10 (Fig. 1F) following treatment with adalimumab. These data suggest there is no direct correlation between ADA binding to the surface of CD4^+^ T cells and IL‐10 expression at day 3.

We also investigated the expression of TNFR1 and TNFR2 on IL‐10 expressing cells. While we were unable to detect TNFR1 on CD4^+^ T cells after culturing cells in the absence or presence of ADA for 3 days (data not shown), we found a significantly increased frequency of TNFR2^+^ cells among IL‐10^+^ compared to IL‐10^−^ cells, however this effect was anti‐TNF treatment independent (Supporting Information Fig. 1J).

### ADA treatment leads to a long‐term induction of IL‐10 expression in CD4^+^ T cells

**Figure 2:**
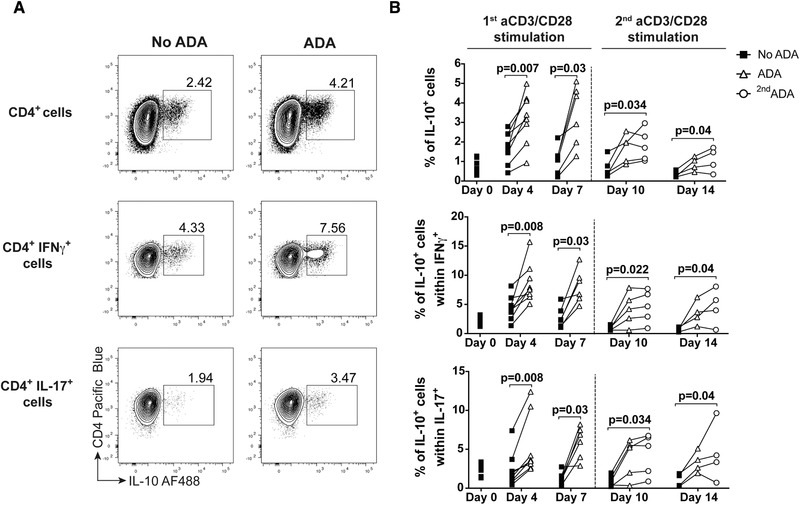
Adalimumab (ADA) treatment has a prolonged effect on IL‐10 production by CD4^+^ T cells. (A) Representative flow cytometry plot showing frequencies for IL‐10 in CD4^+^ T cells at day 4 of stimulation with aCD3/CD28 mAb, in the absence (left) or presence (right) of ADA, in either total CD4^+^ cells (top row) or within either IFN‐γ^+^ (middle row) or IL‐17^+^ (bottom row) cells; (B) cumulative plots showing the frequencies of populations from (A) at day 0, 4, and 7 of stimulation with aCD3/CD28 mAb, in the presence (open triangle) or absence (filled square) of ADA. At day 7, cells received a second round of aCD3/CD28 mAb stimulation and some cells received a second dose of ADA (open circle). Cumulative data from six independent experiments using *n* = 8 distinct donors (data for each time points not available for all donors). Data analysed by either paired Wilcoxon (day 4 and day 7) or Friedman multiple comparisons (day 10 and day 14) tests. Only significant *p*‐values are reported.

To determine whether the effect of ADA treatment on IL‐10 expression in the different populations of CD4^+^ T cells persists over time, we cultured the cells for up to two rounds of aCD3/CD28 mAb stimulation in the absence or presence of ADA and determined IL‐10^+^ cell frequencies by flow cytometry. We observed a significant increase in IL‐10^+^ cell frequencies in ADA‐treated cells for up to 7 days, including in IFN‐γ− and IL‐17‐expressing populations (Fig. 2A and B, and Supporting Information Fig. 2A and B). Following a second round of aCD3/CD28 mAb stimulation, ADA‐treated cells still maintained higher frequencies of IL‐10^+^ cells up to day 14, compared to the control group. Furthermore, a second dose of ADA at restimulation did not further increase IL‐10 frequencies, compared to cells that received ADA at the beginning of cell culture only (Fig. 2B). Finally, we found no significant difference in cell death upon culture of CD4^+^ T cells with ADA over time (Supporting Information Fig. 2C). These data suggest that ADA treatment leads to a long‐term induction of IL‐10 expression in CD4^+^ T cells without inducing cell death.

### In vitro ADA treatment delays CD4^+^ T cell activation, maturation, and proliferation

**Figure 3:**
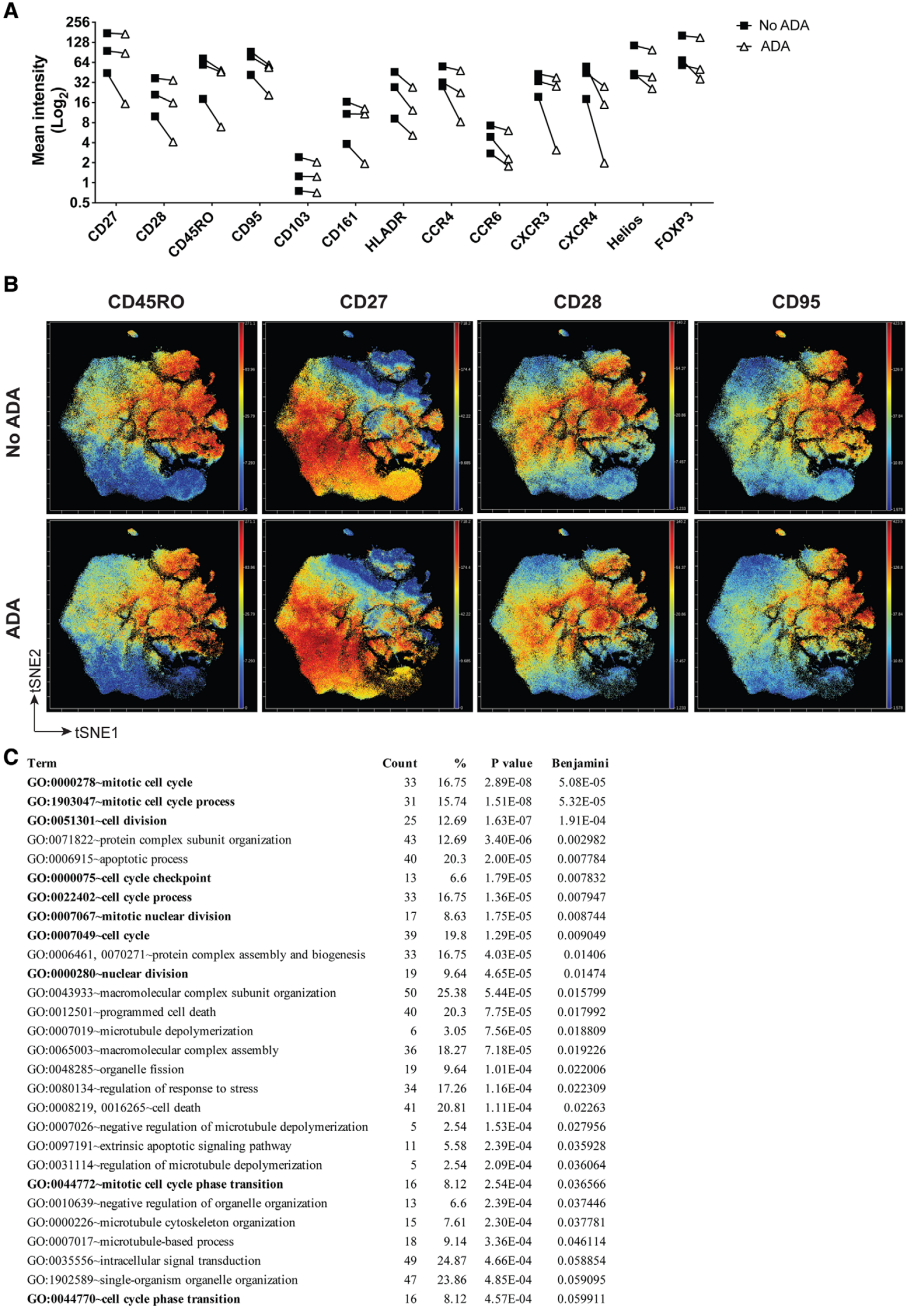
Adalimumab (ADA) treatment leads to negative modulation of multiple CD4^+^ T cell markers. (A) Cumulative plot showing the mean intensity of the markers, as evaluated by CyTOF, commonly downregulated by ADA treatment in CD4^+^ T cells stimulated for 3 days with aCD3/CD28 mAb (results from three independent experiments using *n* = 3 distinct donors); (B) representative viSNE plots of CD4^+^ T cells stimulated for 3 days with aCD3/CD28 mAb in the absence (top row) or presence (bottom row) of ADA; cells were clustered using surface and intracellular markers. Shown are heatmaps for expression of indicated markers (results from three independent experiments using *n* = 3 distinct donors); (C) list of Gene Ontology (GO)‐FAT Biological Processes, subjected to enrichment analysis, from commonly differentially expressed genes (*q* ≤ 0.05) between two differential gene lists generated from microarray data of CD4^+^ T cells stimulated for 3 days with anti‐CD3 mAb and monocytes (2:1 ratio) in the presence or absence of ADA and then sorted for IL‐17 secreting (“Th17”, *n* = 9 donors) or IFN‐γ secreting (“Th1”, *n* = 8 donors) CD4^+^ T cells, representing all genes significantly up‐ or down‐regulated upon TNF‐blockade at the 5% false discovery rate (FDR). Data from five independent experiments. Shown are the number of genes involved in the term (count), percentage of involved genes within the total number of genes (%), Modified Fisher's Exact *p* value, EASE Score (P value), and Benjamini corrected *p* value (Benjamini). The top 30 of 349 total GO terms (ordered by Benjamini corrected *p* value) are shown.

To determine the effect of ADA treatment on early T cell phenotypic changes, we performed CyTOF analysis of CD4^+^ T cells stimulated for 3 days in the absence or presence of ADA, without PMA/Ionomycin. Although there was heterogeneity in cellular phenotype among donors following stimulation, 13 of the 28 markers included in the analysis were commonly downregulated by ADA (Fig. 3A and Supporting Information Fig. 3A). Of these markers, a number were associated with cellular activation and maturation including CD45RO, CD27, CD28, and CD95 (Fig. 3A and B).

In addition, we analysed previously generated gene expression profiling datasets of CD4^+^ T cells cultured in the absence or presence of ADA, which were then sorted for IL‐17‐secreting (Th17) or IFN‐γ‐secreting (Th1) cells. Comparison of the two datasets revealed that 220 genes were commonly regulated by TNF‐blockade: 85 up‐regulated and 128 down‐regulated genes in both Th1 and Th17 cells, and seven genes that were differentially regulated in Th17 vs Th1 cells (Supporting Information Fig. 3B). We performed Gene Ontology (GO)‐FAT Biological Process enrichment analysis on the commonly differentially expressed (with q ≤ 0.05) genes and found that within the top 30 GO terms revealed by our analysis, 10 were associated with cell cycle and division (Fig. 3C), suggesting an effect of ADA on genes associated with these pathways. These data thus pointed to ADA acting as a modulator of cellular activation, maturation, and proliferation of CD4^+^ T cells.

**Figure 4:**
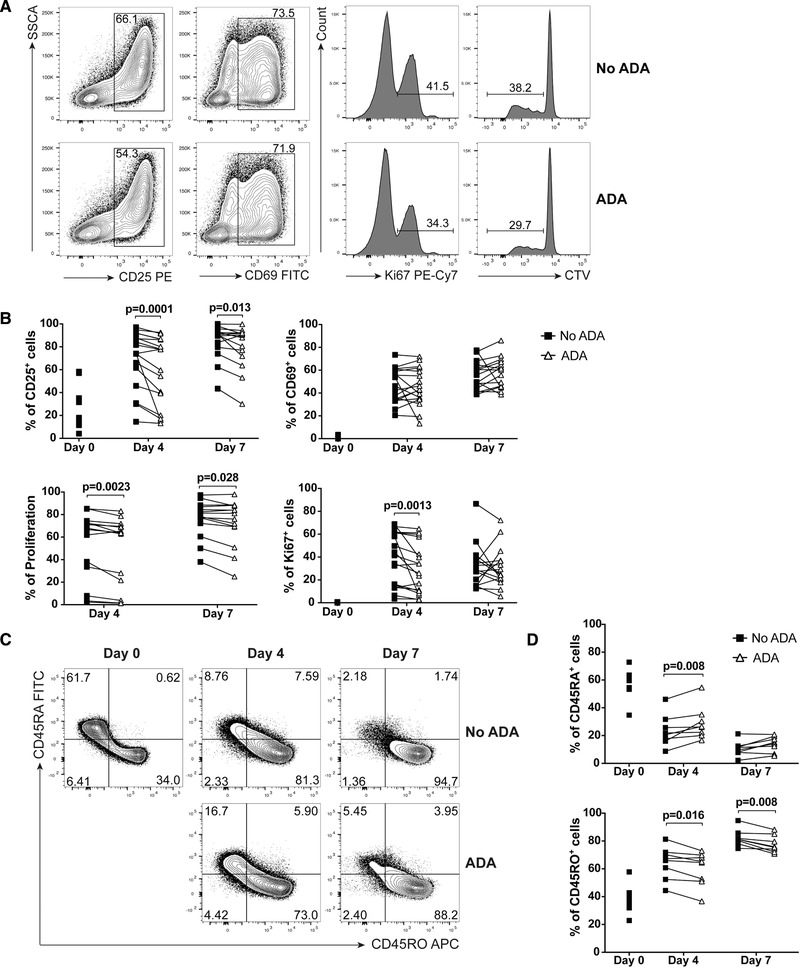
Adalimumab (ADA) treatment leads to delayed activation, proliferation and maturation of CD4^+^ T cells. (A, B) Representative flow cytometry plots (A, day 4) and cumulative data (B) showing percentages of CD25^+^, CD69^+^, proliferating and Ki67^+^ CD4^+^ T cells at day 0, day 4, and day 7 post stimulation with aCD3/CD28 mAb in the absence (filled square) or presence of ADA (open triangle). Data from ten independent experiments using *n* = 14–17 donors; (C, D) representative flow cytometry plots (C) and cumulative data (D) showing percentage of CD45RA^+^ and CD45RO^+^ CD4^+^ T cells at day 0, day 4, and day 7 post stimulation with aCD3/CD28 mAb in the absence (filled square) or presence of ADA (open triangle). Data from four independent experiments, using *n* = 8 donors. All data analysed by Wilcoxon paired test (day 4 and day 7). Significant *p*‐values are reported.

To directly test this hypothesis, we stimulated CD4^+^ T cells with aCD3/CD28 mAb for 7 days in the absence or presence of adalimumab and evaluated by flow cytometry the changes in expression of activation markers CD25 and CD69 as well as proliferation, as measured by expression of Ki67 and CellTrace Violet dye dilution (Fig. 4A and B). ADA treatment led to a significant decrease in the frequency of CD25^+^ cells at day 4. By day 7, the decrease in CD25^+^ cells was less pronounced, suggesting this effect might be due to delayed activation rather than blocked activation. The frequency of CD69^+^ cells, an early activation marker, was not consistently higher or lower in ADA treated cells, at either day 4 or day 7. ADA treatment also resulted in a small but significant decrease in T‐cell proliferation, as determined by CellTrace Violet dye dilution at both day 4 and day 7, and Ki67 expression at day 4.

Changes in activation and proliferation following anti‐TNF treatment could lead to a variation in how CD4^+^ T cells mature and differentiate. Indeed, upon anti‐TNF treatment, a significantly higher proportion of naïve CD4^+^CD45RA^+^CD45RO^−^ cells was present at day 4, but not day 7, in samples treated with ADA. Concomitantly, a significantly lower percentage of memory CD4^+^CD45RA^−^CD45RO^+^ cells was observed in the presence of ADA, at both day 4 and day 7 (Fig. 4C and D).

### ADA treatment does not confer suppressive ability to CD4^+^ T cells

**Figure 5:**
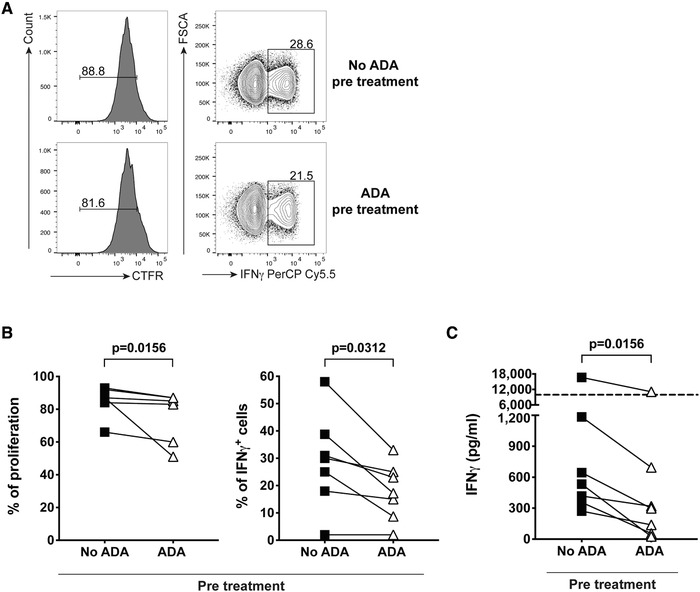
Adalimumab (ADA) pre‐treatment leads to minor hyporesponsiveness of CD4^+^ T cells. (A, B) Representative flow cytometry plots (A) and cumulative plots (B) for proliferation and IFN‐γ staining of CD4^+^ T cells pretreated for 4 days with aCD3/CD28 mAb in the absence (filled square) or presence (open triangle) of ADA and then re‐stimulated for 7 days with aCD3/CD28 mAb stimulation; (C) cumulative plot showing IFN‐γ  secretion from B. Data from five independent experiments using *n* = 7 donors and analysed by Wilcoxon paired test. Significant *p*‐values and lower/upper limits of detection (dashed lines) for cytokine quantification are reported.

We investigated whether preconditioning of CD4^+^ T cells with anti‐TNF mAb could confer anergy or suppressive ability to the cells. We first assessed whether ADA pretreatment rendered the cells hyporesponsive to restimulation. To this end, CD4^+^ T cells were initially cultured with aCD3/CD28 mAb for 4 days in the absence (no ADA pretreatment) or presence (ADA pretreatment) of adalimumab; the cells were then washed and rested for two days, followed by 7 days of stimulation to test their proliferative response and IFNγ expression. CD4^+^ T cells pretreated with ADA showed a small, but consistent decrease in proliferation as well as a significant reduction in IFN‐γ^+^ frequencies and secretion upon restimulation, compared to cells that were pretreated without ADA (Fig. 5).

**Figure 6:**
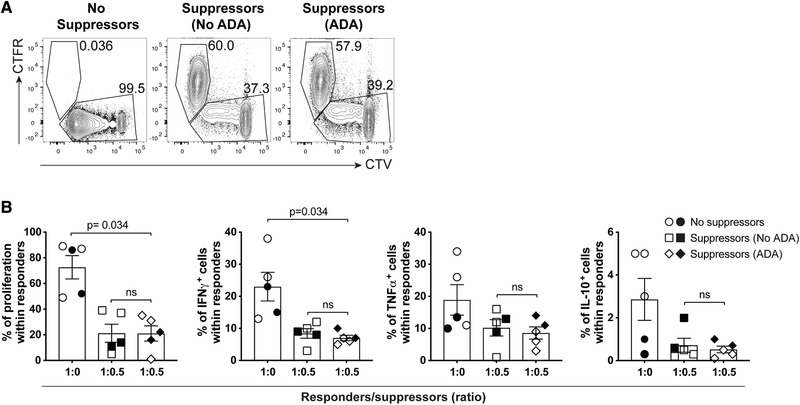
Adalimumab (ADA) pretreatment does not confer a suppressive phenotype to CD4^+^ T cells. (A) Representative flow cytometry plots showing gating strategy for co‐cultures containing CellTrace Far Red (CTFR) labelled CD4^+^ T suppressor cells pretreated for 4 or 7 days with aCD3/CD28 mAb in the absence (No ADA) or presence (ADA) of ADA and CellTrace Violet (CTV) labelled bulk CD4^+^ or CD4^+^CD25^−^ responder cells after they have been co‐cultured for 4 or 7 days with aCD3/CD28 mAb; (B) cumulative plots showing percentages of proliferating, IFN‐γ^+^, TNF‐α^+^ and IL‐10^+^ cells within either bulk CD4^+^ (filled symbols) or CD4^+^CD25^−^ (open symbols) responder cells. Bars show mean ± SEM of results from three independent experiments using *n* = 5 donors and analysed by Friedman multiple comparisons test. Significant *p*‐values are reported.

Next, we determined whether pre‐treatment with ADA led cells to become suppressive by co‐culturing ADA pretreated or control pretreated CellTrace Far Red (CTFR) labelled cells with responder CellTrace Violet (CTV) labelled cells (Fig. 6A). To this end, CD4^+^ T cells were initially cultured with aCD3/CD28 mAb for 4 or 7 days in the absence (no ADA pre‐treatment) or presence (ADA pre‐treatment) of adalimumab; the cells were then washed and rested for two days, followed by testing of the proliferative response and cytokine production of the responder cells. A significant reduction in proliferative capacity and IFN‐γ^+^ cell frequencies was observed in responder CD4^+^ T cells that were co‐cultured with CD4^+^ T cells that had previously been treated with ADA (Suppressors (ADA)), however a similar effect was observed when responder cells were co‐cultured with CD4^+^ T cells treated without ADA (Suppressors (No ADA)) (Fig. 6B). Thus, there was no differential effect in the suppressive activity of cells that had been pretreated with or without ADA and depletion of Tregs (open symbols) did not alter this result. Furthermore, no significant differences were observed in the frequencies of TNF‐α^+^ or IL‐10^+^ responder T cells upon co‐culture (Fig. 6B).

### ADA pretreated CD4^+^ T cells do not differentially modulate monocyte activation

**Figure 7:**
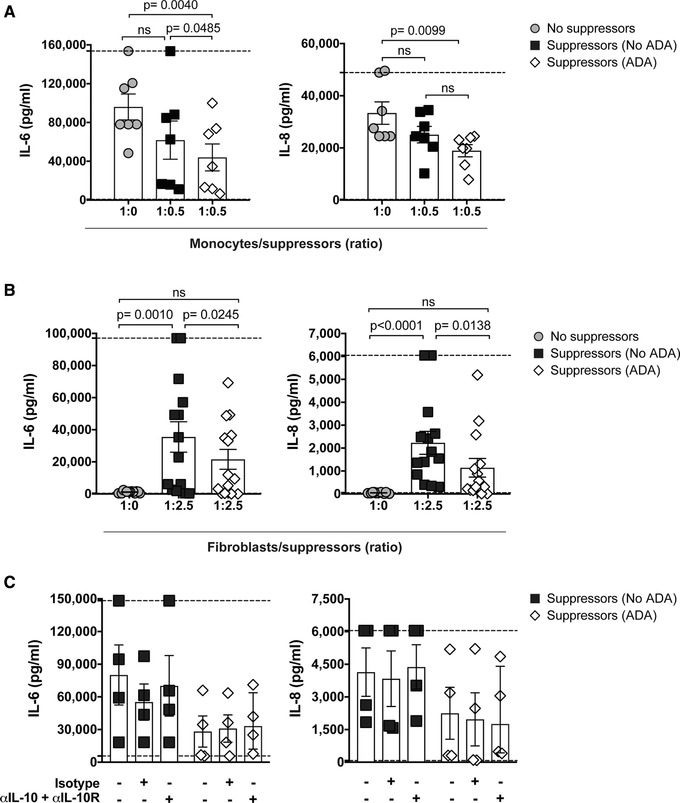
Adalimumab (ADA) pretreatment of CD4^+^ T cells leads to reduced inflammatory phenotype of fibroblasts post co‐culture. (A, B) Cumulative plots showing supernatant concentrations of IL‐6 and IL‐8 after either 40 h co‐culture of monocytes in presence of LPS (A, *n* = 7) or after 3 days co‐culture of fibroblasts (B, *n* = 14) with either no CD4^+^ T cells (grey circles), No ADA (black squares) or ADA (open diamonds) pretreated suppressors; (C) cumulative plots showing supernatant concentrations of IL‐6 and IL‐8 post 3 days co‐culture of fibroblasts with either No ADA (black squares) or ADA (open diamonds) pretreated suppressors in the absence or presence of blocking anti‐IL‐10 and anti‐IL10R antibodies or isotype controls (*n* = 4). Bars show mean ± SEM of results from four (A), five (B), and two (C) independent experiments all analysed by pairwise Friedman multiple comparisons test. Significant *p*‐values and lower/upper limits of detection (dashed lines) for cytokine quantification are reported.

Since pretreatment of CD4^+^ T cells with ADA did not lead to the acquisition of a specific T cell‐suppressive phenotype, we evaluated whether anti‐TNF‐treated T cells might affect monocyte phenotype and function. To this end, we cultured CD4^+^ T cells for 3 days with aCD3/CD28 mAb in the absence or presence of ADA; cells were washed and rested, and then added into co‐cultures with monocytes for 40 h in the absence (for phenotypic analysis) or presence (for cytokine analysis) of LPS. No significant differences in monocyte expression of HLA‐DR, CD80, CD86, CD40, or CD163 were observed when monocytes were co‐cultured with ADA pretreated T cells compared to control pretreated cells (Supporting Information Fig. 4). In terms of LPS‐induced cytokine production, co‐culture with ADA pre‐treated T cells led to a significant reduction in monocytic IL‐6 and IL‐8 production compared to the no T cell control; when compared to co‐culture with control‐treated T cells, LPS‐induced production of IL‐6, but not IL‐8, was slightly lower (Fig. 7A). Collectively, these data show that a specific regulatory effect of ADA‐treated CD4^+^ T cells on monocyte activation is seen only in terms of IL‐6 production in response to LPS.

### ADA pretreated CD4^+^ T cells induce lower pro‐inflammatory cytokine production by fibroblasts

Finally, we sought to investigate whether ADA pretreated T cells could affect RA fibroblast function. To this end, we stimulated CD4^+^ T cells for 3 days in the absence or presence of adalimumab and then washed, rested, and co‐cultured the cells with RA fibroblasts for 3 days before evaluating cytokine production post co‐culture. Co‐culture of RA fibroblasts and T cells was required to obtain detectable amounts of IL‐6 and IL‐8, independent of the pretreatment condition (Fig. 7B). However, co‐culture of fibroblasts with ADA pretreated cells led to significantly lower production of IL‐6 and IL‐8 compared to co‐culture with cells pretreated without ADA (Fig. 7B). Unexpectedly, this effect was independent of IL‐10 since the addition of blocking antibodies to IL‐10 and IL‐10R did not reverse the reduction in pro‐inflammatory cytokines (Fig. 7C).

## Discussion

Expanding on our previous work 16, the single cell CyTOF analysis presented here demonstrates that adalimumab treatment affects multiple CD4^+^ T cell subsets leading to both a boost and expansion of IL‐10 producing cells, including in cells actively expressing proinflammatory cytokines like IFN‐γ and IL‐17. We found that the effect of adalimumab on IL‐10 production persisted over our 2‐week culture period, however a repeat dose of adalimumab did not significantly enhance the production of IL‐10 compared to cells receiving only one dose. We found no association between IL‐10 producing cells and binding of adalimumab to the CD4^+^ T cell surface, suggesting that the effect of anti‐TNF on modulation of IL‐10 expression occurs independently of binding to transmembrane TNF, which is expressed at very low levels on the surface of CD4^+^ T cells.

The increased expression of the anti‐inflammatory cytokine IL‐10, paired with the negative modulation of multiple markers following stimulation, led us to evaluate whether TNF signalling inhibition affected CD4 T‐cell activation. While a downregulation of activation marker CD25 was found following anti‐TNF treatment, variation in CD69 expression did not show a consistent trend. CD69, however, is an early and short‐term activation marker, compared to the “late” activation marker CD25 21. Given we only assessed T‐cell activation at days 4 and 7, it is possible that any initial modulatory effects on CD69 expression had normalized by then. Previous studies also showed that anti‐TNF leads to decreased T‐cell activation, as demonstrated by variation of CD25 expression, in IBD 22, 23, ulcerative colitis 24, psoriasis 23, and RA 25, while the effect on CD69 was more variable 23, 25. Some of these studies showed decreased proliferation following TNF‐blockade 22, 24, while one study reported an increased proliferative response in patients upon TNF‐blockade 23. These studies however evaluated the effect of anti‐TNF on T‐cell activation and proliferation in the context of whole PBMC cultures or mixed lymphocyte reactions, where the TNF inhibition affects both the T cell and APC compartments. Indeed, CD4^+^ T cells co‐cultured with monocytes from RA patients on TNF‐inhibitors showed decreased proliferation 26, with another study attributing this effect to the induction of regulatory macrophages in an Fc dependent manner 20. Our data show that anti‐TNF drugs can delay both T cell activation and proliferation in an APC independent manner. Furthermore, we found that adalimumab treatment led to a short‐term skewing of the balance between naïve and memory CD4^+^ T cells towards the former, which normalised over time. This delayed but eventually normalising effect on maturation would be consistent with the reported lack of variation in percentages of circulating memory T cell subsets in patients receiving anti‐TNF therapy for 1–3 months 23. Finally, we did not find a significant difference in cell death upon culture with ADA over time, which is consistent with previously published data that anti‐TNF does not induce apoptosis in T cells or MLR 20. Our data therefore suggest that TNF‐blockade can exert direct delaying effects on CD4^+^ T‐cell activation, proliferation, and maturation, but does not affect the long‐term viability and ability of T cells to respond to stimulation.

We have previously demonstrated that anti‐TNF mediated IL‐10 induction in T cells is independent of Tregs 15, 16. There is no consensus on how TNF‐blockade affects Tregs; studies have reported an increase in expression of Foxp3 and/or suppressive function of Tregs after neutralization of TNF 27, 28, 29, 30, 31, while other studies showed that TNF signaling through TNFRII promoted Treg expansion and function 32, 33 and therefore anti‐TNF can decrease Treg activity 34. Our data show that culturing CD4^+^ T cells in the presence of adalimumab led to a small decrease in Foxp3 and Helios expression accompanied by a limited reduction in proliferation. We found a more pronounced effect on IFN‐γ expression and secretion upon re‐stimulation, but did not find evidence for profound T cell anergy. Furthermore, CD4^+^ T cells that were pretreated with adalimumab, despite the increased IL‐10 production, did not show a significantly greater suppressive ability, compared to control‐treated cells, when co‐cultured with responder T cells. These data therefore suggest that TNF‐blockade does not confer a Treg‐like phenotype or suppressive function to CD4^+^ T cells.

We showed that anti‐TNF pretreatment of CD4^+^ T cells did not dramatically affect the capacity of the cells to modulate the phenotype and function of monocytes, except for a small reduction in LPS‐induced IL‐6 production. Other studies have shown that anti‐TNF may affect the interaction between monocytes and T cells leading to a more tolerogenic phenotype. It was shown that adalimumab promoted binding between monocytes of RA patients, which expresses high levels of mTNF, and TNF‐RII‐expressing Tregs, leading to the expansion of the latter 35. It was also shown that monocyte binding of the Fc region of anti‐TNF antibodies promoted the generation of regulatory M2‐like CD206^+^ macrophages, post co‐culture with CD4^+^ T cells 20. In our co‐culture setting, where anti‐TNF is limited to the CD4^+^ T cell pretreatment stage, we found no overall variation in the expression of costimulatory molecules on the monocytes and only a limited effect on LPS‐induced IL‐6 production. These data suggest that anti‐TNF preconditioning of CD4^+^ T cells alone is insufficient to significantly modulate monocyte phenotype in vitro.

Synovial fibroblasts are particularly important in the context of RA given their role in the active invasion of articular cartilage and damage mediated by release of proinflammatory and matrix‐degrading mediators (reviewed in ref. 36). TNF‐α is a strong inducer of fibroblast activation and is a key driver of inflammation and joint destruction; systemic over‐expression of human TNF in vivo is sufficient to initiate chronic synovium inflammation, cartilage destruction, and bone erosion, which can be ameliorated by treatment with anti‐TNF 37. Interestingly, our data showed that anti‐TNF pre‐treated CD4^+^ T cells have a reduced ability to induce the production of proinflammatory mediators by RA fibroblasts, which is independent of IL‐10. This finding suggests that anti‐TNF pre‐conditioning could prime CD4^+^ T cells to regulate fibroblast‐driven inflammation, even after TNF inhibition is no longer present.

Further work is required to investigate how TNF inhibition therapy restrains the immune response in RA and in particular how modulation of IL‐10 expression contributes to this immunoregulation. Furthermore, insight into the molecular mechanisms via which TNF‐blockade operates, both in maintaining IL‐10 expression and regulating cellular activation and function, could identify potential targets for novel therapeutic strategies.

## Materials and methods

### Cell isolation

Peripheral blood samples were obtained from healthy adult volunteers. PBMCs were isolated by density gradient centrifugation using Lymphoprep (Axis‐Shield, Oslo, Norway). CD4^+^ T cells and CD14^+^ monocytes were isolated by MACS using the CD4^+^ T cell Isolation Kit II and CD14 MicroBeads, respectively (Miltenyi Biotec, Bergisch‐Gladbach, Germany). Average purities were 96% for both CD4^+^ T cells and CD14^+^ monocytes. For isolation of CD4^+^CD25^−^ T cells, CD4^+^ T cells were subsequently depleted of CD25^+^ cells using CD25 MicroBeads II (Miltenyi Biotec). The study was approved by the Bromley Research Ethics Committee (06/Q0705/20), and written informed consent was obtained from all participants.

### CD4^+^ T cell culture

Cells were cultured at 37°C with 5% CO_2_ in RPMI 1640 medium (Gibco) supplemented with 10% heat‐inactivated fetal bovine serum (Sigma) and 1% penicillin, streptomycin and l‐glutamine (all from Gibco). MACS‐isolated CD4^+^ T cells were stimulated at 10^6^/mL with 1.25 µg/mL of coated anti‐CD3 (clone OKT3; BD Biosciences) and 1 µg/mL of soluble anti‐CD28 (clone CD8.2; BD Biosciences) in the absence or presence of 1 µg/mL of adalimumab (ADA, Abbott Laboratories, Chicago, USA) for up to 7 days. For subsequent rounds of stimulation, cells were washed, counted, and restimulated as described above.

### Hyporesponsiveness assay

Bulk CD4^+^ T cells were cultured and stimulated as described above for 4 days in the absence or presence of adalimumab (1 µg/mL). Cells were washed and rested for 48 h at 37°C. Cells were then labelled with CTFR or CTV; both at 1 µM, LifeTechnologies (Carlsbad, USA, following manufacturer's instructions) and stimulated for 7 days with aCD3/CD28 mAb stimulation, as above. Proliferation and IFN‐γ production were measured by flow cytometry and ELISA.

### T cell suppression assay

Bulk CD4^+^ T cells were cultured and stimulated as described above for either 4 or 7 days in the absence or presence of adalimumab (1 µg/mL). Cells were washed and rested for 48 h at 37°C. Suppressor cells were then labelled with CTFR (1 µM) and co‐cultured with CTV (1 µM; both LifeTechnologies, Carlsbad, USA) bulk CD4^+^ or CD4^+^CD25^−^ T cells, as responders, at 1:0.5 (Responders:Suppressors) ratio for 4 or 7 days with either standard aCD3/CD28 mAb stimulation or aCD3/CD28 beads (GIBCO) at a 1:40 bead to cell ratio. Proliferation and IFN‐γ frequencies were measured in responder cells post co‐culture by flow cytometry.

### Monocyte modulation assay

Bulk CD4^+^ T cells to be used for monocyte modulation were cultured and stimulated as described above for 3 days in the absence or presence of adalimumab (1 µg/mL). Cells were washed and rested for 24 h. The pre‐cultured CD4^+^ T cells were then co‐cultured with autologous CD14^+^ monocytes for 40 h at 1:0.5 ratio (CD14^+^:CD4^+^) with soluble aCD3 mAb stimulation (100 ng/mL); phenotype was assessed by flow cytometry. For evaluation of cytokine production, LPS (50 ng/mL) was added for the duration of the co‐culture and supernatants collected at 40 h.

### Fibroblast modulation assay

Bulk CD4^+^ T cells were cultured and stimulated as described above for 7 days in the absence or presence of adalimumab (1 µg/mL), with supplementation of 20 U/mL (Peprotech) of IL‐2 on day 4. Cells were washed and rested for 24 h at 37°C. Fibroblasts generated from RA patients' knee replacement synovial tissue were seeded in 96‐well flat bottom plates at a density of 10 000/well in DMEM, supplemented with 10% FCS, 1% penicillin/streptomycin, 2% l‐glutamine (all from GIBCO), and 1 µg/mL Amphotericin B/Fungizone (GIBCO) and allowed to adhere for 24 h. Twenty‐five thousand pre‐cultured CD4^+^ T cells were added to the fibroblasts and cultured in DMEM for 3 days with soluble aCD3 mAb (100 ng/mL) with or without 10 µg/mL of anti‐IL‐10 (JES3‐19F1, Rat IgG2a, Biolegend) and anti‐IL‐10R (3F9, Rat IgG2a, Biolegend) blocking antibodies or matching isotype control Ab (RTK2758, Rat IgG2a, Biolegend). Supernatants were collected post co‐culture.

### Flow cytometry

To assess proliferation, CD4^+^ T cells were stained with either 1 µM of CTV or CTFR (both from Life Technologies), before stimulation and culture. To assess intracellular cytokine expression after cell culture, cells were stimulated for 3 h in the presence of PMA (50 ng/mL, Sigma‐Aldrich), ionomycin (750 ng/mL, Sigma‐Aldrich), and GolgiStop (BD Biosciences); GolgiPlug (BD Biosciences) was additionally added for complete blocking of TNF export when measuring expression of mTNF. Cells were labelled with a fixable viability dye (LIVE/DEAD fixable dead cell stains, ThermoFisher Scientific). Cells were subsequently washed and stained extracellularly, followed by fixation with 2% PFA (paraformaldehyde, Sigma‐Aldrich), permeabilization using the FOXP3 perm buffer (BioLegend). Cells were stained with various combinations of the following fluorescently conjugated antibodies (see Supporting Information Table 1). Stained cells were acquired using a FACSCantoII or LSRFortessa (BD Biosciences); in most experiments, 100 000 T cell events were recorded. All flow cytometry data were analysed using FlowJo software (version 10, Tree Star, Ashland, USA). Representative CD4^+^ T cell gating strategy is shown in Supporting Information Fig. 2. Guidelines for the use of flow cytometry in immunological studies were adhered to 38.

### CyTOF staining and analysis

CD4^+^ T cells were isolated using MACS isolation and 3 × 10^6^ cells labelled with metal conjugated antibodies for extracellular, intracellular, or intranuclear staining. Supporting Information Table 2 shows the full list of both antibody panels (panel 1 for samples stimulated without PMA/Ionomycin; panel 2 for samples stimulated for 3 h with PMA/Ionomycin before staining). CyTOF‐2 mass cytometer (Fluidigm) was used for data acquisition and beads (Ce140) were used for normalization 39. Data were initially processed and analysed using Cytobank 39. At least 350 000 events from the CD4^+^ or CD4^+^CD45RO^+^ gates (Supporting Information Fig. 1A) were equally sampled from all individuals to perform automated clustering. Mass‐cytometry complex data was analysed using viSNE, in combination with SPADE, to identify distinct subpopulations 17, 18 using the following parameters: for cytokine production evaluation: CCR6, CD62L, CD45RA, CD95, IL‐4, CCR4, CD161, CD103, TNFα, TIM3, PD1, Helios, IFNγ, CCR7, Tbet, CTLA4, CD27, TGFβ, IL‐17A, CD45RO, IL‐2, GATA3, IL‐10, Foxp3, TIGIT, CXCR4, HLA‐DR, LAG3, CD127; for marker modulation by ADA treatment: CCR6, CD62L, CD45RA, CD95, ICOS, CCR4, CD161, CD103, CD69, TIM3, PD1, Helios, CXCR3, CCR7, Tbet, CTLA4, CD27, CD28, CD45RO, GATA3, IL‐10, CD40L, Foxp3, TIGIT, CXCR4, HLA‐DR, LAG3, CD127. viSNE and SPADE plots were generated using Cytobank (CA, USA). To quantify similarities/differences among the nodes generated by SPADE and identify the subsets among the clustered T cells, MEM for each identified population was calculated 19. Medians of the markers were hyperbolic arcsine (asinh) transformed (cofactor = 5). Where indicated in the figure legend, figures were overlaid for demonstration purposes.

### Enzyme‐linked immunosorbent assay (ELISA)

IFN‐γ, IL‐6, and IL‐8 were measured in cell culture supernatants using the ELISA MAX kits (BioLegend), according to manufacturer's instructions. Microwell absorbance was read at 450 nm using a Spark 10M microplate reader (TECAN, Austria).

### Gene ontology analysis

Gene ontology analysis was performed on two datasets: one based on CD4^+^ T cells stimulated for 3 days with anti‐CD3 mAb and monocytes (2:1 ratio) in the presence or absence of ADA and then FACS sorted for IL‐17 secreting (Th17) cells (GSE51540, as described in ref. 15), and one based on CD45RO^+^ enriched CD4^+^ T cells (CD45RO microbeads, Miltenyi), cultured as described above and then sorted for IFN‐γ secreting CD4^+^ T (Th1) cells (GSE65054). RNA extraction, purification, integrity analysis, and gene expression microarrays were carried out at Novo Nordisk A/S (Måløv, Denmark). Samples were run on a Human Genome U219 Array plate (Affymetrix). Chips were scanned and gene expression data were normalized using the Robust Multi‐array Average (RMA) algorithm and the Bioconductor package “Affy” (http://www.bioconductor.org). Custom CDF (chip definition file) from brainarray.mbni.med.umich.edu was used. Gene expression profiling analysis was performed using Qlucore Omics Explorer 3.0 software (Qlucore AB, Lund, Sweden). Two differential gene lists were generated from each set of microarray data, representing all genes up/down‐regulated by TNF‐blockade in IL‐17‐secreting (“Th17”) or IFN‐γ‐secreting (“Th1”) CD4^+^ T cells at 5% false discovery rate (FDR). Ensembl gene IDs from each gene list were compared using the online platform Venny (http://bioinfogp.cnb.csic.es/tools/venny/). Commonly differentially expressed (q ≤ 0.05) genes were subjected to Gene Ontology (GO)‐FAT Biological Process enrichment analysis using DAVID.

### Statistical analysis

Statistical testing was performed with GraphPad Prism 7.0 (GraphPad, San Diego, CA, USA). Data sets were tested using the appropriate non‐parametric test as indicated in figure legends. *p*‐values < 0.05 were considered statistically significant.

## Conflict of Interest

K.S.F. is an employee of Novo Nordisk A/S. The remaining authors declare no commercial or financial conflict of interest.

**Abbreviations**
ADA: adalimumab
CTFR: CellTrace Far Red
CTV: CellTrace Violet
MEM: marker enrichment modelling
RA: rheumatoid arthritis

## Supporting information

FigureS1
