# Supplementary material for: Anti‐TNF treatment negatively regulates human CD4+ T‐cell activation and maturation in vitro, but does not confer an anergic or suppressive phenotype
Source: Eur J Immunol. 2019 Dec 3;50(3):445–58. doi: 10.1002/eji.201948190 (PMC7079027; doi:10.1002/eji.201948190)
Supplement: Supplementary file 1 — FigureS1 [file EJI-50-445-s001.pdf]

European Journal of Immunology

Supporting Information

for

DOI 10.1002/eji.201948190

Giovanni A. M. Povoleri, Sylvine Lalnunhlimi, Kathryn J. A. Steel, Shweta Agrawal, Aoife M. O'Byrne, Michael Ridley, Shahram Kordasti, Klaus S. Frederiksen, Ceri A. Roberts and Leonie S. Taams

Anti-TNF treatment negatively regulates human $CD4^{+}$ T-cell activation and maturation in vitro, but does not confer an anergic or suppressive phenotype

© 2019 The Authors. *European Journal of Immunology* published by WILEY-VCH Verlag GmbH & Co. KGaA, Weinheim.

Figure S1

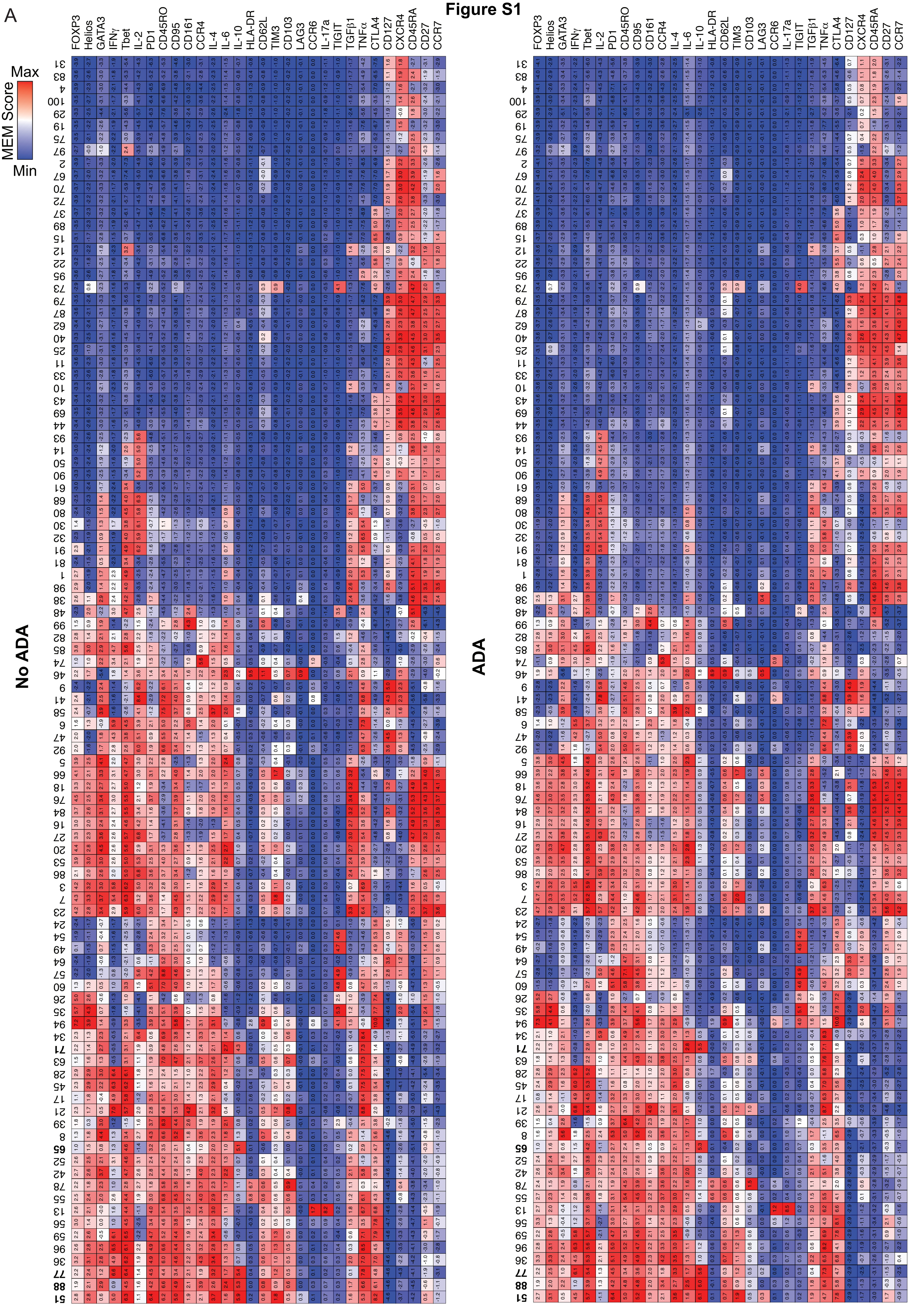
A
MEM Score
Max
Min
FOXP3
Helios
GATA3
IFNγ
Tbet
IL-2
PD1
CD45RO
CD95
CD161
CCR4
IL-4
IL-6
IL-10
HLA-DR
CD62L
TIM3
CD103
LAG3
CCR6
IL-17a
TIGIT
TGFβ1
TNFα
CTLA4
CD127
CXCR4
CD45RA
CD27
CCR7
No ADA
ADA

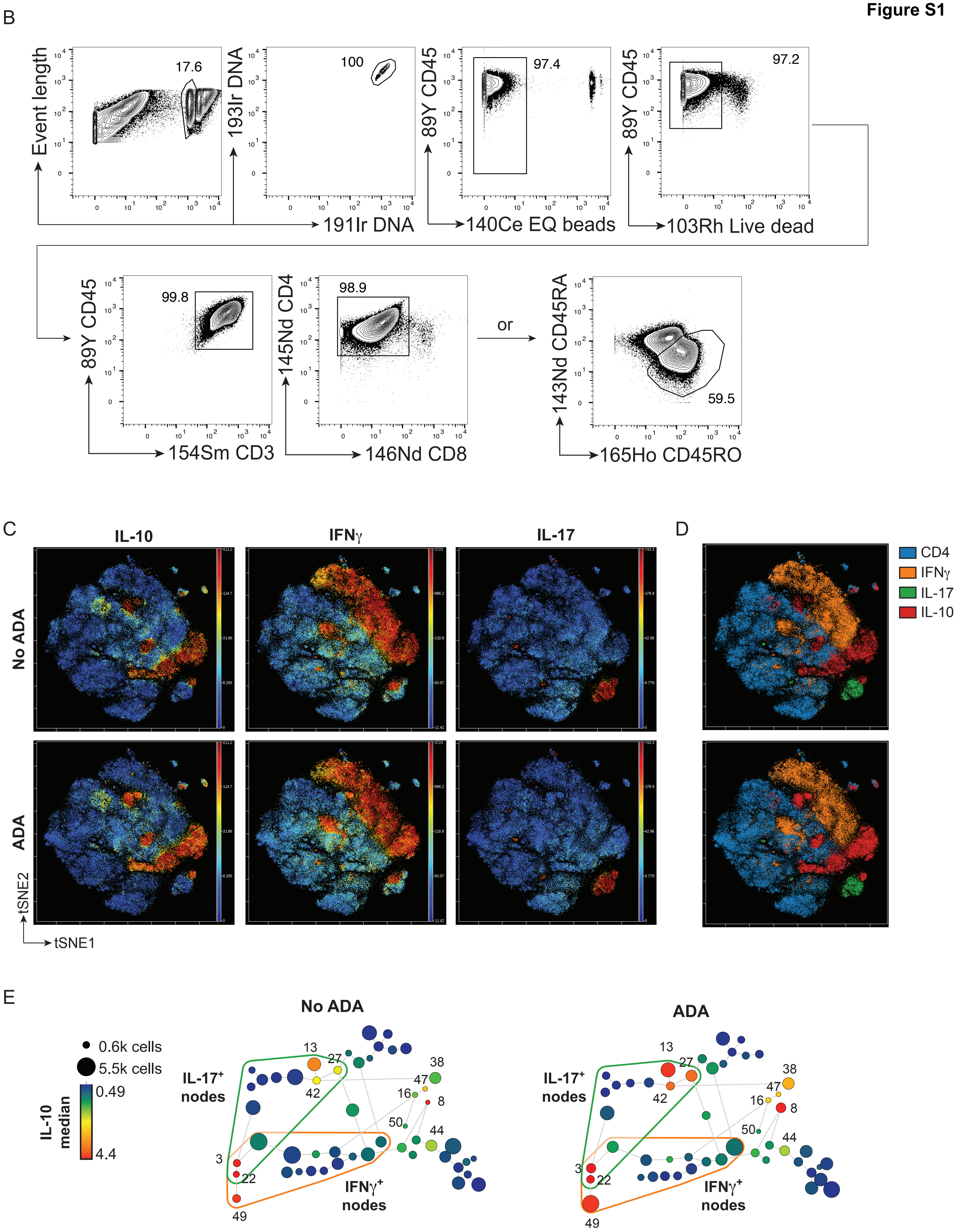
B
Event length
191Ir DNA
193Ir DNA
140Ce EQ beads
89Y CD45
103Rh Live dead
154Sm CD3
145Nd CD4
146Nd CD8
143Nd CD45RA
165Ho CD45RO
17.6
100
97.4
97.2
99.8
98.9
59.5
or
C
IL-10
IFNγ
IL-17
No ADA
ADA
tSNE2
tSNE1
D
CD4
IFNγ
IL-17
IL-10
E
No ADA
ADA
0.6k cells
5.5k cells
IL-10 median
0.49
4.4
IL-17+ nodes
IFNγ+ nodes

F

Figure S1

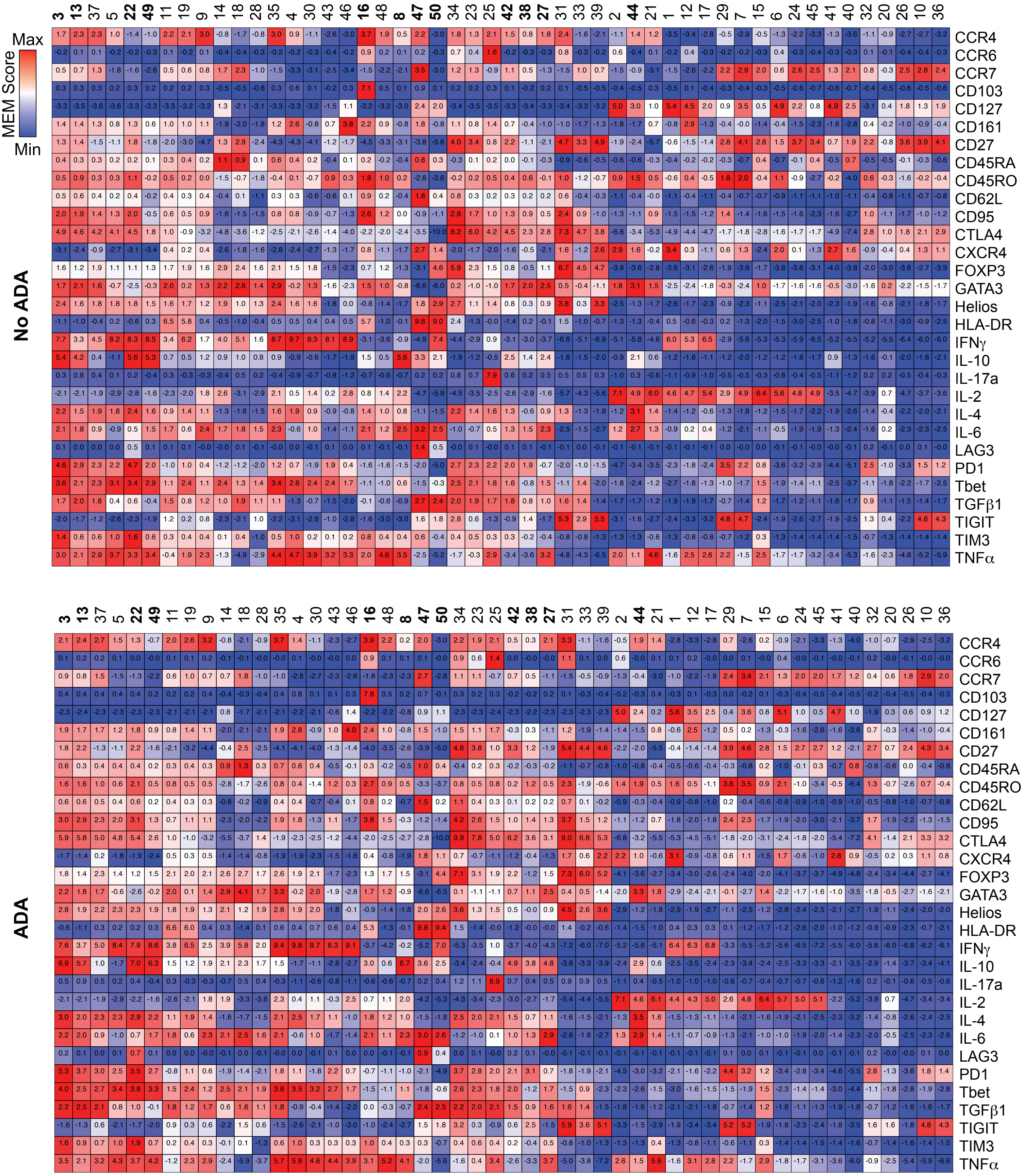
MEM Score
Max
Min
No ADA
ADA
3 13 37 5 22 49 11 19 9 14 18 28 35 4 30 43 46 16 48 8 47 50 34 23 25 42 38 27 31 33 39 2 44 21 1 12 17 29 7 15 6 24 45 41 40 32 20 26 10 36
CCR4
CCR6
CCR7
CD103
CD127
CD161
CD27
CD45RA
CD45RO
CD62L
CD95
CTLA4
CXCR4
FOXP3
GATA3
Helios
HLA-DR
IFNγ
IL-10
IL-17a
IL-2
IL-4
IL-6
LAG3
PD1
Tbet
TGFβ1
TIGIT
TIM3
TNFα

Figure S1

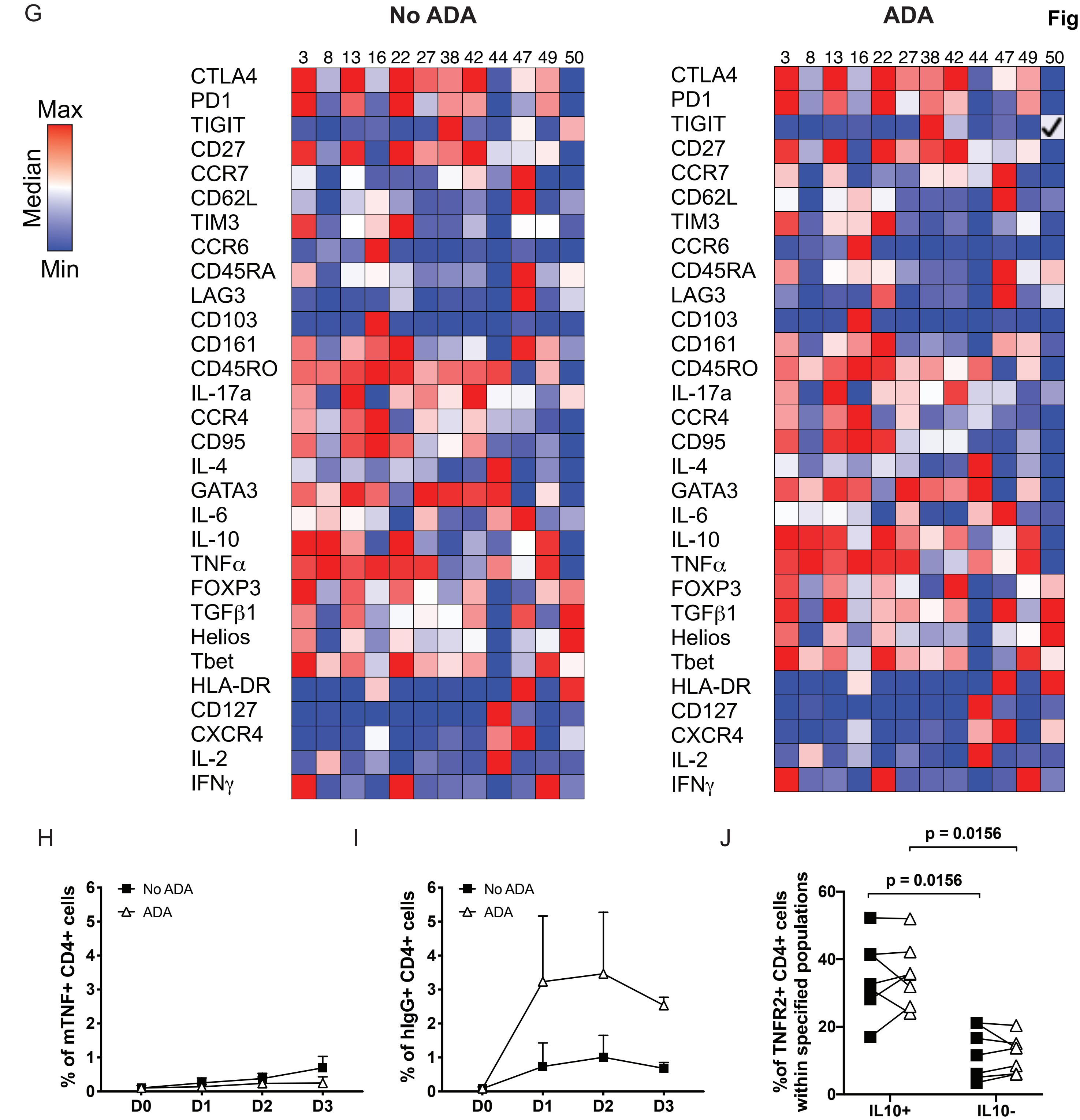

Supplementary Figure 1. (A) Heatmap showing MEM scores of the 100 nodes identified by SPADE analysis shown in Figure 1C. Nodes are displayed as columns and markers as rows. Enrichment score for each marker is reported. (B) Representative plot demonstrating the gating strategy for CyTOF analysis of CD4+ or CD4+CD45RO+ T-cells pre-tSNE. In this example, purified CD4+ T-cells were stimulated for 3 days with aCD3/CD28, showing gating strategy to gate intact cells (191Ir+ DNA stain), cells (191Ir+193Ir+), no beads (89Y CD45+140Ce-), live (89Y CD45+ 103Rh-), CD3+ (89Y CD45+ 154Sm CD3+) and CD4+ (145Nd CD4+ 146Nd CD8-) or CD4+ CD45RO+ (145Nd CD4+ 143Nd CD45RA- 165Ho CD45RO+) T-cells. (C) viSNE plots of CD4+ T-cells stimulated for 3 days with aCD3/CD28 mAb, and 3 hours of PMA/Ionomycin, in the absence (top row) or presence (bottom row) of ADA; CD4+CD45RO+ cells were clustered using surface and intracellular markers. Shown are heatmaps for expression of indicated markers. (D) Overlay of IL-10+ (red), IFNγ+ (orange) and IL-17+ (green) T-cells on viSNE map of CD4+ T-cells (blue) showing the overlap of cells either expressing IL-10 alone or in combination with other cytokines in the absence or presence of ADA. (E) 2D minimum spanning tree (SPADE) derived from viSNE analysis showing population nodes of CD4+ T-cells either cultured in the absence (left panel) or presence (right panel) of ADA and clustered as CD4+CD45RO+ T-cells. Node size represents cell number, and colour IL-10 median intensity. Grouped together are IL-17+ (circled in green) and IFNγ+ (circled in orange) nodes. Numbered nodes represent IL-10+ nodes identified from S1F. (F) Heatmap showing MEM scores of the 50 nodes identified by SPADE analysis shown in Figure S1E. Nodes are displayed as columns and markers as rows. Enrichment score for each marker is reported. (G) Correlation matrix showing median expression of markers from IL-10+ nodes from S1E and S1F. One donor from three independent experiments is shown. (H-I) Cumulative plots showing the frequencies of mTNF+ CD4+ T cells (H) or hIgG+ (I) CD4+ T-cells either ex vivo (D0), or at day 1, day 2 or day 3 of stimulation with aCD3/CD28 mAb, in the absence (filled square) or presence (open triangle) of ADA. Bars show mean and SD. Data from 2 independent experiments using 3 donors in total. (J) Cumulative plot showing the frequencies of TNF-RII+ CD4+ T cells among IL-10+ and IL-10- cells after 3 days stimulation with aCD3/CD28 mAb, and 3 hours of PMA/Ionomycin, in the absence (filled square) or presence (open triangle) of ADA. Data from four independent experiments using n=7 different donors. Data analysed by two-tailed paired Wilcoxon test.

Figure S2

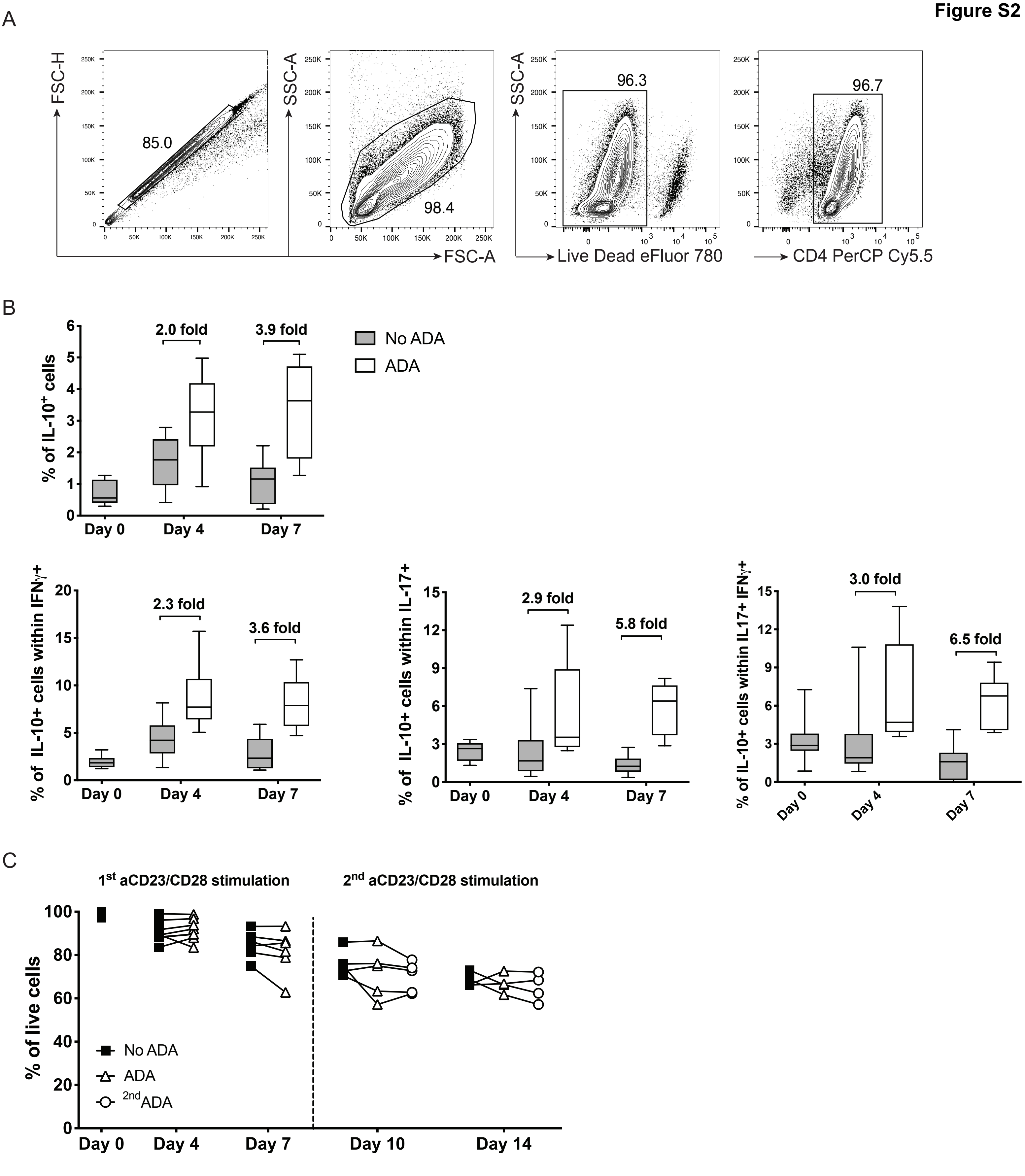

Supplementary Figure 2. (A) Representative plot demonstrating the gating strategy for the flow cytometric analysis of CD4+ T-cells. In this example, purified CD4+ T-cells were stimulated for 3 days with aCD3/CD28, showing gating strategy to gate single cells (FSC-H x FSC-A), lymphocytes (SSC-A x FSC-A), live cells (SSCA x Live Dead) and CD4+ T-cells (SSC-A x CD4). (B) cumulative plots showing the average proportion of IL-10+, IL-10+ within IFNγ+, IL-10+ within IL-17+ and IL10+ within IFNγ+IL17+ cells (Figure 1A) at day 0, 4 and 7 of stimulation with aCD3/CD28 mAb, in the presence or absence of ADA. Data from six independent experiments. Fold change between No ADA and ADA treated samples reported. (C) Cumulative plot showing the frequencies of live cells (as measured by live-dead cell staining) at day 0, 4 and 7 of stimulation with aCD3/CD28 mAb, in the absence (filled square) or presence (open triangle) of ADA. At day 7, cells received a second round aCD3/CD28 mAb stimulation and some cells received a second dose of ADA (open circle) (n=4-8). Data from six independent experiments analysed by either paired Wilcoxon (day 4 and day 7) or Friedman multiple comparisons (day 10 and day 14) tests. No significant differences were found.

Figure S3

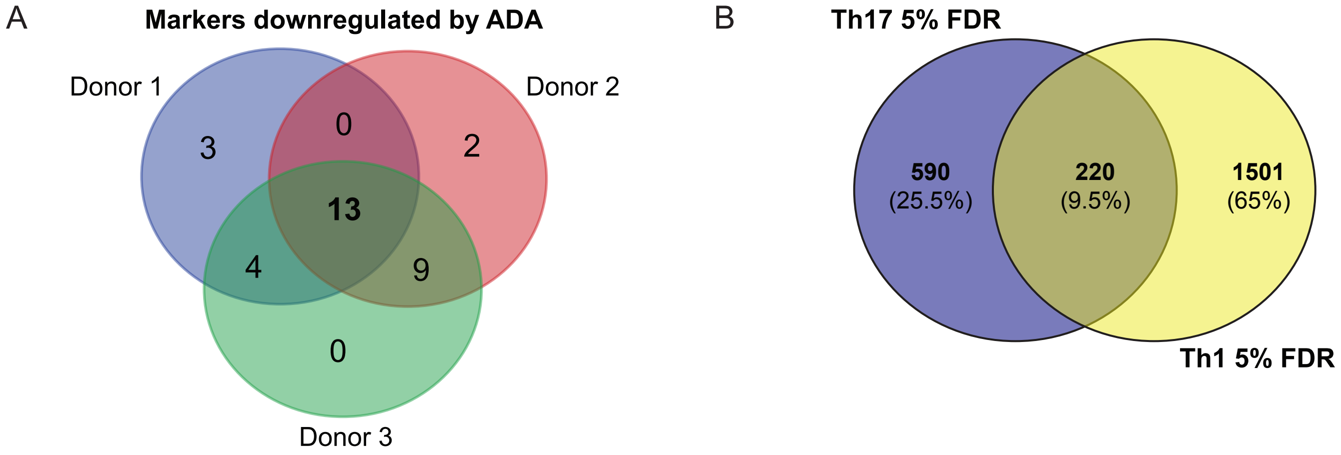

Supplementary Figure 3. (A) Venn diagram showing the number of markers, as evaluated by CyTOF, that were downregulated by ADA treatment in CD4+ T-cells stimulated for 3 days with aCD3/CD28 mAb. (B) Venn diagram showing the overlap between two differential gene lists generated from microarray data, representing all genes regulated upon TNF-blockade in IL-17-secreting (“Th17”, n=9) or IFNγ-secreting (“Th1”, n=8) CD4+ T-cells at the 5% false discovery rate (FDR).

Figure S4

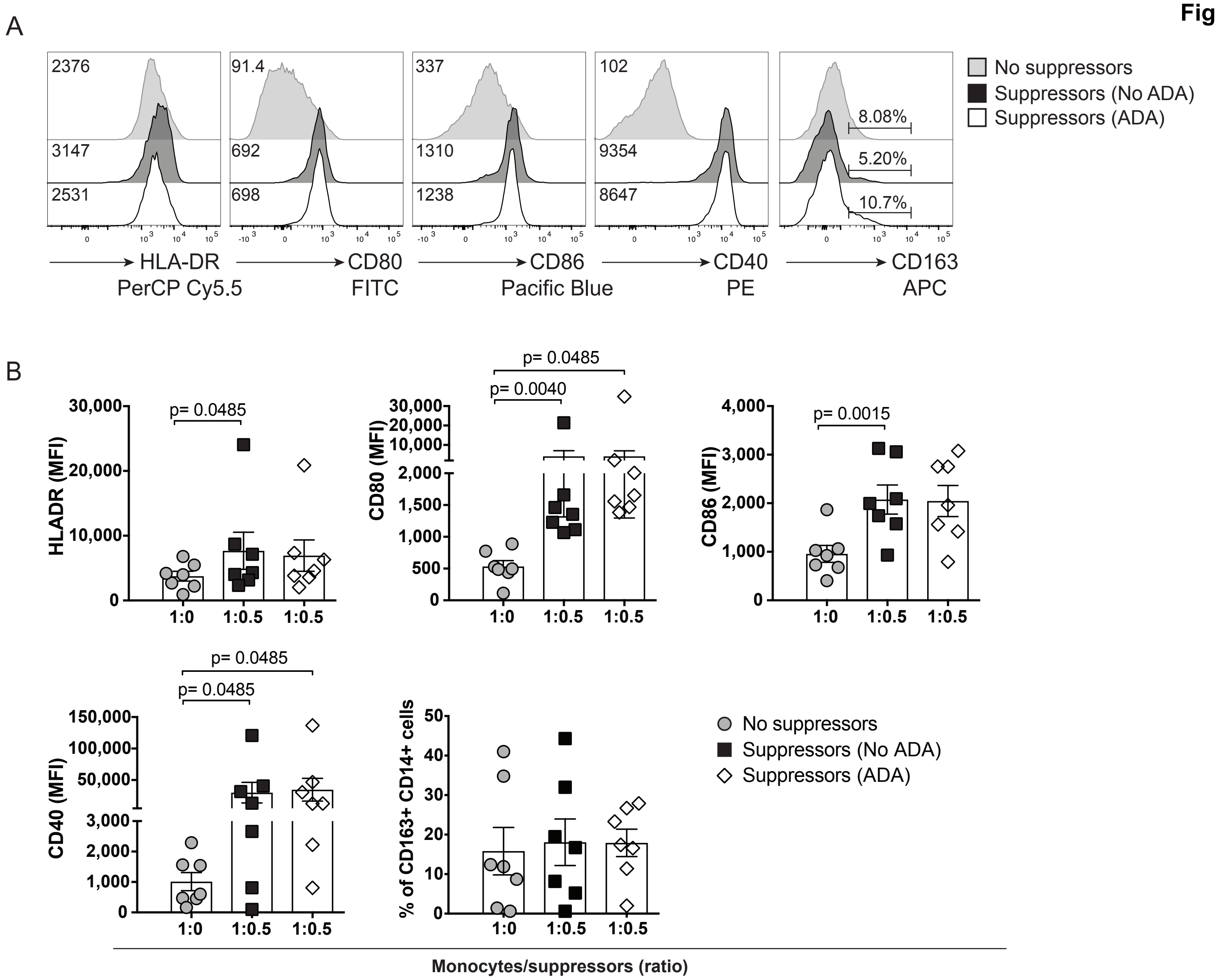

Supplementary Figure 4. ADA pre-treated CD4+ T-cells do not differentially affect monocyte phenotype post co-culture. CD4+ T-cells were cultured for 3 days with anti-CD3/CD28 mAb in the absence or presence of adalimumab (1µg/ml). Cells were washed and rested for 24hrs. The pre-cultured CD4+ T-cells were then co-cultured with autologous CD14+ monocytes for 40 hours at 1:0.5 ratio (CD14+:CD4+) with soluble aCD3 mAb stimulation (100 ng/ml); phenotype was assessed by flow cytometry. (A, B) Representative flow cytometry plots (A) and cumulative data (B) showing MFI of HLA-DR, CD80, CD86, CD40 and percentage of CD163+ monocytes post 40 hours co-culture with either no CD4+ T-cells suppressors (grey histograms and symbols), No ADA (black histograms and symbols) or ADA (clear histograms and open symbols) pre-treated suppressors (n=7). Data from three independent experiments using multiple donors analysed by Friedman multiple comparisons test.

Reactivity	Target	Clone	Supplier
Human	CD4	SK3	BioLegend
Human	IL-10	JES3-9D7	BioLegend
Human	IL-17A	BL168	BioLegend
Human	IFNγ	S.B3	BioLegend
Human	TNFa	MAb11	BioLegend
Human	CD25	M-A251	BioLegend
Human	CD69	FN50	BioLegend
Human	Ki67	Ki-67	BioLegend
Human	CD45RA	HI100	BioLegend
Human	CD45RA	UCHL1	BioLegend
Human	IgG Fc	HP6017	BioLegend
Human	CD120b	3G7A02	BioLegend

Supplementary Table 1. Flow cytometry antibody list.

Channel	Target	Clone	Supplier	Panel
89	CD45	HI30	Fluidigm	1 and 2
141	CD196 (CCR6)	G034E3	Fluidigm	1 and 2
142	CD62L	DREG-56	BioLegend	1 and 2
143	CD45RA	HI100	Fluidigm	1 and 2
145	CD4	RPA-T4	Fluidigm	1 and 2
146	CD8a	SK1	Fluidigm	1 and 2
147	CD95	DX2	BioLegend	1 and 2
148	ICOS	C398.4A	Fluidigm	1
148	IL-4	MP4-25D2	BioLegend	2
149	CCR4	205410	Fluidigm	1 and 2
150	CD161	HP-3G10	BioLegend	1 and 2
151	CD103	Ber-ACT8	BioLegend	1 and 2
151	CD123	6H6	Fluidigm	1 and 2
152	CD69	FN50	BioLegend	1
152	TNFa	Mab11	Fluidigm	2
153	TIM3	F38-2E2	BioLegend	1 and 2
154	CD3	UCHT1	Fluidigm	1 and 2
155	PD1	EH12.2H7	Fluidigm	1 and 2
156	Helios	ICFC	BioLegend	1 and 2
158	CXCR3	G025H7	BioLegend	1
158	IFNg	B27	Fluidigm	2
159	CCR7	G043H7	Fluidigm	1 and 2
160	Tbet	4B10	Fluidigm	1 and 2
161	CTLA4	L3D10	eBioscience	1 and 2
162	CD27	O323	BioLegend	1 and 2
163	TGFb	TW46H10	Fluidigm	2
164	CD28	CD28.2	BioLegend	1
164	IL-17A	N49-653	Fluidigm	2
165	CD45RO	UCHL1	Fluidigm	1 and 2
166	IL-2	MQ1-17H12	BioLegend	2
167	GATA3	TWAJ	Fluidigm	1 and 2
168	CD40L	24-31	Fluidigm	1
168	IL-6	MQ2-13A5	BioLegend	2
170	IL-10	JES3-9D7	BioLegend	2
171	FOXP3	150D	BioLegend	1 and 2
171	FOXP3	259D	BioLegend	1 and 2
171	FOXP3	PCH101	eBioscience	1 and 2
172	TIGIT	MBSA43	eBioscience	1 and 2
173	CXCR4	12G5	Fluidigm	1 and 2
174	HLA-DR	L243	Fluidigm	1 and 2
175	LAG3	11C3C65	Fluidigm	1 and 2
176	CD127	A019D5	Fluidigm	1 and 2

Supplementary Table 2. CyTOF antibody list for panel 1 (no PMA/Io stimulation) and panel 2 (PMA/Io stimulation).
